# The self-organization of plant microtubules inside the cell volume yields their cortical localization, stable alignment, and sensitivity to external cues

**DOI:** 10.1371/journal.pcbi.1006011

**Published:** 2018-02-20

**Authors:** Vincent Mirabet, Pawel Krupinski, Olivier Hamant, Elliot M. Meyerowitz, Henrik Jönsson, Arezki Boudaoud

**Affiliations:** 1 Reproduction et Développement des Plantes, Univ. Lyon, ENS de Lyon, UCB Lyon 1, CNRS, INRA, F-69364 Lyon, France; 2 Sainsbury Laboratory, University of Cambridge, Cambridge, United Kingdom; 3 Computational Biology and Biological Physics Group, Department of Theoretical Physics, Lund University, Lund, Sweden; 4 Division of Biology and Biological Engineering, California Institute of Technology, Pasadena, California, United States of America; 5 Howard Hughes Medical Institute, California Institute of Technology, Pasadena, California, United States of America; 6 Department of Applied Mathematics and Theoretical Physics, University of Cambridge, Cambridge, United Kingdom; University of British Columbia, CANADA

## Abstract

Many cell functions rely on the ability of microtubules to self-organize as complex networks. In plants, cortical microtubules are essential to determine cell shape as they guide the deposition of cellulose microfibrils, and thus control mechanical anisotropy of the cell wall. Here we analyze how, in turn, cell shape may influence microtubule behavior. Building upon previous models that confined microtubules to the cell surface, we introduce an agent model of microtubules enclosed in a three-dimensional volume. We show that the microtubule network has spontaneous aligned configurations that could explain many experimental observations without resorting to specific regulation. In particular, we find that the preferred cortical localization of microtubules emerges from directional persistence of the microtubules, and their interactions with each other and with the stiff wall. We also identify microtubule parameters that seem relatively insensitive to cell shape, such as length or number. In contrast, microtubule array anisotropy depends on local curvature of the cell surface and global orientation follows robustly the longest axis of the cell. Lastly, we find that geometric cues may be overcome, as the network is capable of reorienting toward weak external directional cues. Altogether our simulations show that the microtubule network is a good transducer of weak external polarity, while at the same time, easily reaching stable global configurations.

## Author summary

Plants exhibit an astonishing diversity in architecture and morphology. A key to such diversity is the ability of their cells to coordinate and grow to reach a broad spectrum of sizes and shapes. Cell growth in plants is guided by the microtubule cytoskeleton. Here, we seek to understand how microtubules self-organize close to the cell surface. We build upon previous two-dimensional models and we consider microtubules as lines growing in three dimensions, accounting for interactions between microtubules or between microtubules and the cell surface. We show that microtubule arrays are able to adapt to various cell shapes and to reorient in response to external signals. Altogether, our results help to understand how the microtubule cytoskeleton contributes to the diversity of plant shapes and to how these shapes adapt to environment.

## Introduction

Despite their amazing diversity in shapes, biological organisms share some common structural components at the cellular level. Among those, one of the best conserved proteins across eukaryotes, tubulin, assembles into protofilaments, which in turn form 25 nm nanotubes known as microtubules, usually made of 13 protofilaments. The network of microtubules is highly labile and can reshape itself in a matter of minutes. In plants, microtubules form superstructures before (the preprophase band), during (the spindle) and after (the phragmoplast) cell division. Plant microtubules also form dense and organized arrays at the periphery of the cell during interphase [1] and these arrays are known as cortical microtubules (CMTs). The behavior of CMTs has been studied extensively at the molecular level [2]. One of their main functions is to guide the trajectory of the transmembrane cellulose synthase complex and thus to bias the orientation of cellulose microfibrils in the wall. This in turn impacts the mechanical anisotropy of the cell wall and controls growth direction [3–6]. This function explains why most mutants impaired in microtubule-associated proteins exhibit strong morphological defects [7].

Whereas this provides a clear picture on how microtubules impact cell shape, in turn how cell shape impacts microtubule behavior has been less explored. There is evidence that microtubule orientation depends on cell shape [8–10], with microtubules being mostly transverse to the longest axis, but this might require specific regulation because microtubules orient along the longer axis of the confining domain in vitro [11]. There is also evidence that shape-derived mechanical stress can bias cellulose deposition, possibly through microtubule orientation towards the direction of maximal tension, both at the tissue and single cell scales [8, 12–21]. The molecular mechanism behind remains largely unknown. Finally, cortical microtubules orientation may change in response to signals such as blue light or hormones, see for instance [17, 22] and may be oriented by the hydrodynamic forces due to cytoplasmic streaming [23, 24]. Here we use modeling to explore the relative contributions of cell geometry and external directional cues in the final microtubule organization.

The molecular basis for microtubule dynamics is rather well established. Consistent with the absence of centrosome in land plants, microtubule nucleation is dispersed in plant cells, as it occurs at the cell cortex [25], along existing microtubules during branching events [25–27], and at the nuclear envelope [28]. As they grow, microtubules form stiff and polar structures. They can alternate growth, pause and shrinking at the so-called plus end [29], whereas they mainly shrink or pause at the minus end [30]. The combination of an average shrinkage at the minus end and dynamic instability at the plus end leads to an overall displacement of the microtubule, also called hybrid treadmilling [30], with a dominant contribution of short treadmilling microtubules in the final microtubule organization [31]. The growth of microtubules in persistent directions is the main cause for microtubule encounters.

When one microtubule encounters another microtubule, different outcomes can be observed [9, 32]: if the encounter angle is shallow, zippering can occur, i.e. the growing microtubule bends and continues its polymerization along the encountered microtubule, which leads to the creation of microtubule bundles; if the encounter angle is steep, crossover can occur, i.e. a microtubule polymerizing without deviating its trajectory and crossing over the encountered microtubule; or alternatively catastrophe is triggered, i.e. a rapid plus end shrinkage after contact with the encountered microtubule. Such selective pruning of microtubules may explain how microtubules can form parallel arrays from initially random orientations, and conversely change the net orientation of their arrays over time, through a phase of randomisation [33, 34]. Selective pruning has indeed an essential ingredient of most models for microtubule dynamics [35–44]. The presence or absence of different microtubules associated proteins (MAPs) can modulate the stability of microtubules or their capacity to form bundles and to self-organize. For instance, the microtubule severing protein Katanin accounts for most of the pruning events at crossover sites [45].

The microtubule network is a typical example of a self-organizing system, where properties of individual elements and their interactions induce specific and sometimes counter-intuitive global properties. To predict how regulation at the level of each microtubule can give rise to specific global outcomes, one can resort to computational models. Modeling approaches have been developed, simplifying microtubule interactions by restricting them to the plasma membrane, i.e. a simpler 2D space [40, 46]. In those agent-based models, several microtubule properties were coded and interactions between CMTs, based on these properties, were simulated. The outcome is an emergent network, whose characteristics can be analyzed. For instance, increased microtubule severing was predicted to generate a larger number of free microtubules, more amenable to bundle into aligned arrays [9, 42] and this was observed in experiments [47].

So far, most of the microtubule models have been implemented in a 2D space or with microtubules confined to the surface of the cell. A major outcome of such models was to demonstrate that global orientations of the network can spontaneously emerge from the interactions between microtubules [48]. Many combinations of parameters and behaviors have been studied: instability at the plus end [35, 37], role of zippering [9, 36, 38, 39], nucleation modes [39, 42], and severing [44]. Beyond their differences, a global orientation emerges in most of these combinations suggesting that converging toward a global orientation is a robust feature of microtubule networks. Conversely, the diverse combinations of microtubule properties provide different scenarios for the fine-tuning of the network structure and stability of this emerging behavior.

Some aspects of cell geometry were related to microtubule behavior in certain simulations. Simulations showed how different directional biases in nucleation can induce an ordering of the array toward directions that are correlated to cell geometry [35, 43]. Further, branching nucleation rules can elicit handedness of the global direction of microtubule arrays, provided that the branching is biased toward one direction [41]. Other studies used a simulation space where borders, analog to cell edges, induce more or fewer catastrophe events or are more or less permissive toward microtubule growth [10, 33, 35, 41]. Most studies concluded that a global orientation of microtubules can be correlated to cell face orientations.

The contribution of the third dimension to microtubule behavior has started to be investigated. Computational models for animal systems have focused on 3D considerations but the nucleation hypotheses are too different from that in plants to be transposed directly [49, 50]. Fully 3D models suited for plants are still lacking: almost all existing studies have confined microtubules to surfaces embedded in 3D [10, 11, 41, 51]. In [35], a 2D model was extended into a full 3D model but it did not include cell boundaries, which yielded microtubules distributed over the whole simulated domain, in contrast with the cortical localization of microtubules in planta.

In this paper, we explore the influence of 3D cell shape on the basic properties of a dynamic microtubule network. We do not assume that microtubules are confined to the cell surface; rather, we simulate a closed volume where microtubules are more or less free to grow in all directions. Anchoring to the membrane is not imposed by the model and instead becomes a variable in the model. Using this framework, we investigate to what extent microtubule interaction with the membrane can influence microtubule dynamics. Our study also addresses the relative contributions of cell shape, microtubule interactions, and external directional cues in network organization.

## Results

### A model for interphase microtubules growing at the plasma membrane or within the cytoplasm

**Fig 1:**
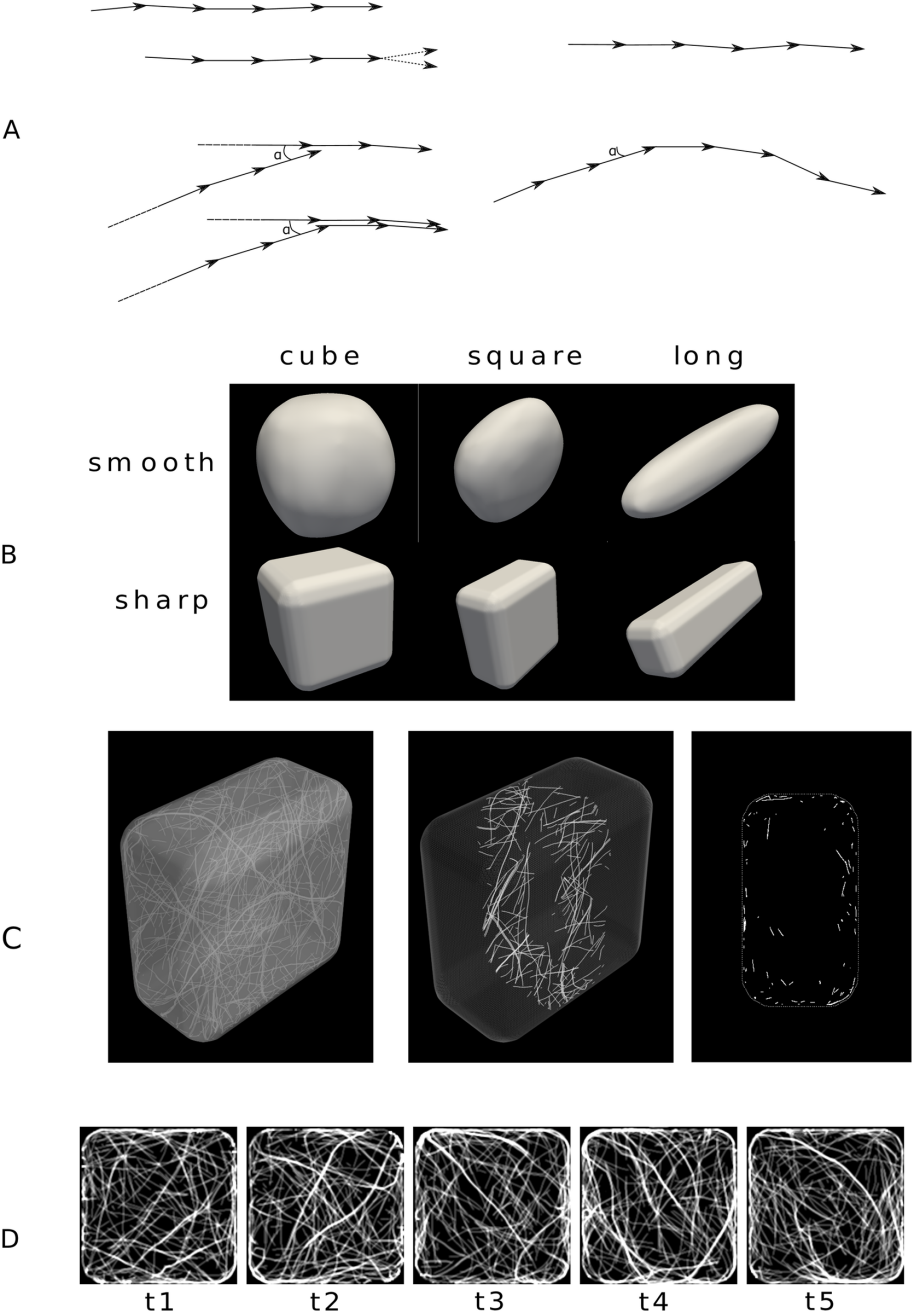
Shapes of simulated cells. (A) Top left: Two time steps of a simulation when the microtubule grows freely inside the cell and simultaneously shrinks from the minus end; at the plus side (arrowhead), the new vector is calculated by adding a small random deviation to the previous vector. Bottom left: Example of a zippering process—When the new microtubule encounters an existing one at a shallow angle (if the angle *a* is smaller than threshold *α*), the new direction of the vectors follows that of the leading microtubule; a steep angle would lead to plus end shrinkage. Top right: Strong anchoring to the plasma membrane—The microtubule grows tangentially to the local plane. Bottom right: Weak anchoring to the membrane—When the microtubule encounters the membrane at a shallow angle (*a* < *α*), it continues tangentially until the random deviation leads it to leave the membrane. (B) Shapes of the envelope of the cell: cube, “square”, and “elongated”. First row: smooth shapes. Second row: sharp shapes. Scales are not respected. (C,D) Example of a simulation in a sharp square with weak anchoring and default parameters (see Methods); the length of the simulation is 50000 timesteps, corresponding to approximately 100 minutes, also see S2 Video. (C) Snapshot showing all microtubules (left), microtubules located in a central slice that is ∼2.4*μ*m-thick (center), and the projection of this slice in 2D (right). (D) Snapshots taken after 10000 timesteps intervals (∼20 min); the pictures show a confocal microscopy-like *z*-projection (see Methods).

Following previous studies, we modelled microtubules as a set of line segments that nucleate, grow, shrink, and interact with each other and with the cell surface represented as a triangular mesh (see Methods for details). Nucleation of the minus end occurs randomly at the surface. Growth occurs from the plus end with a small directional noise that is related to the persistence length of microtubules (Fig 1A). Shrinkage starts randomly at the nucleation site (minus end) and then continues at constant velocity.

A microtubule that encounters a preexisting microtubule either changes direction to that of the preexisting microtubule if the encounter angle is shallow (a process known as zippering, see Fig 1A), and otherwise starts shrinkage from the plus end (“head-on” collision). We considered two types of interaction with the cell surface: strong anchoring, whereby microtubules remain on the surface, as in previous studies [10, 11, 41, 51], and weak anchoring, whereby microtubules are prevented from leaving the cell interior. More specifically, in the case of weak anchoring, the interaction between a growing microtubule and the nearby surface is similar to the interaction between two microtubules: the microtubule encountering the surface at a steep angle starts shrinking, and otherwise starts growing tangentially to the surface (Fig 1A); a microtubule may leave the surface because of the directional noise.

We considered three base shapes: cube, “square” (flattened cube), and “long” (elongated small cube), of dimensions in the order of 10 *μ*m, typical of plant cells. These shapes were smoothed so that the maximal curvature corresponded to a radius of either ∼1 *μ*m (“sharp”) or ∼5 *μ*m (“smooth”), which corresponds to typical radii of curvature measured in root epidermis [10], see Fig 1B. We also considered an ellipsoidal shape when investigating the effects of an external cue. Although these shapes are not fully realistic, they make it easier to disentangle the geometrical parameters influencing microtubule dynamics.

A typical simulation with weak anchoring is given in S1 Video. Corresponding snapshots are shown in Fig 1C and 1D with various 3D and 2D views. A few first observations can be made: the microtubules tend to bundle; a well-defined local orientation appears; microtubules appear to be mostly close to the cortex.

### Microtubules become cortical in a 3D space because of their directional persistence and growth mode

We first considered the effect of the anchoring of the microtubules to the membrane. There are proteins that have been shown to be associated with both microtubules and a plasma membrane component in plants [52]. For instance, CELLULOSE SYNTHASE INTERACTIVE PROTEIN 1 (CSI1) interacts with both CMTs and the cellulose synthase (CESA) complex [6, 53] and CSI1 was also proposed to stabilize the microtubule network [54]. Yet, the influence of CMT-CESA interactions on the microtubule network is still poorly understood. Thus, we took advantage of the 3D nature of our model to study the impact of the anchoring rule to the membrane on the global parameters of the microtubule network.

**Fig 2:**
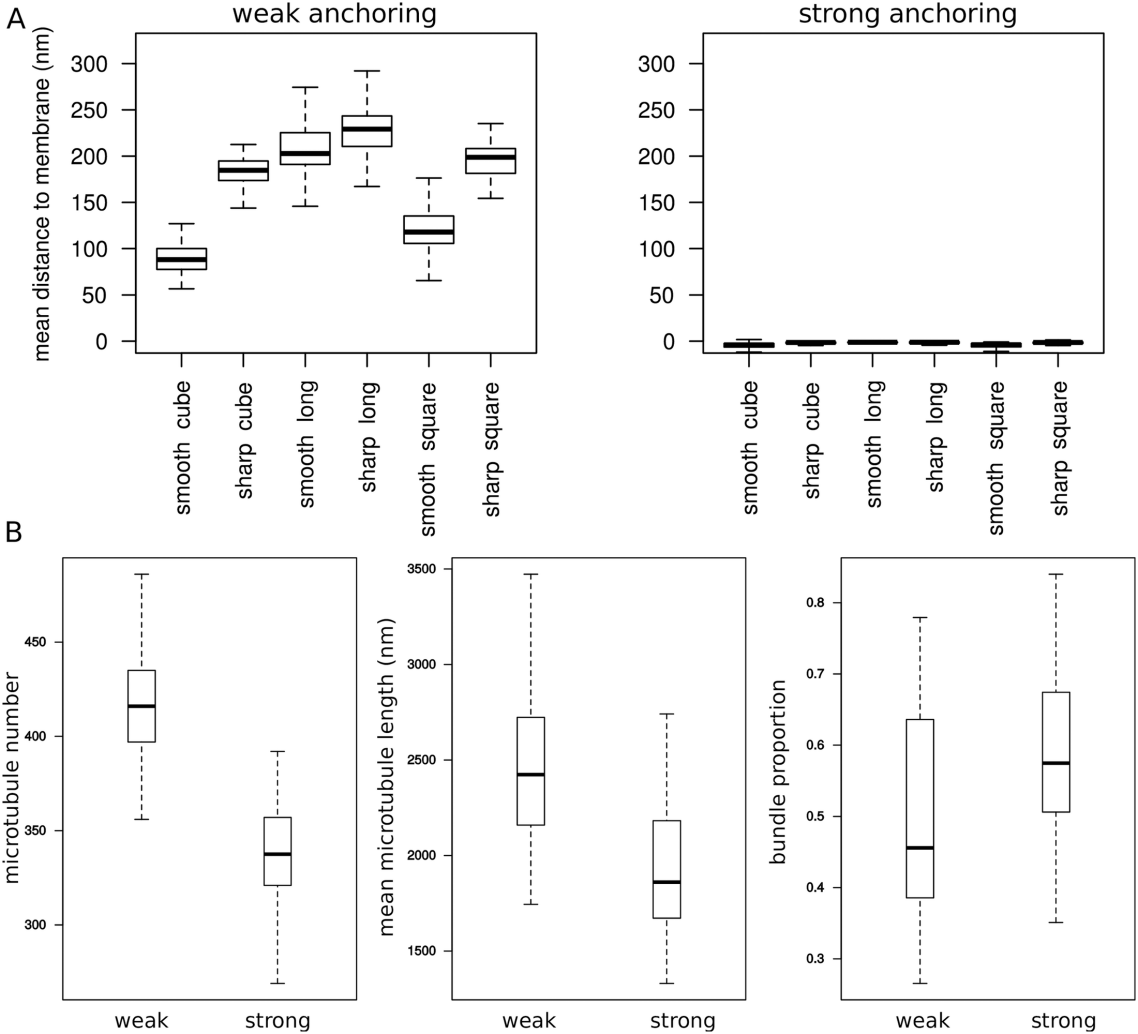
Influence of the anchoring to the membrane. (A) Mean distance of microtubules to the membrane according to cell shape and to weak/strong anchoring to the membrane. (B) Effect of anchoring on three properties of the microtubule network (data for all shapes pooled together). Left: total number of microtubules. Middle: mean microtubule length. Right: proportion of tubulin in bundles. Model parameters have the default values (see Methods).

We investigated the microtubule dynamics when the anchoring to the membrane is weak. As a reference case, we also considered strong anchoring, whereby microtubule are constrained to grow on the membrane, as if putative anchoring proteins were highly concentrated. As expected, strong interactions led to a surface-localized cortical zone with microtubules trajectories embedded in the plane parallel to the mesh (Fig 2A).

In the case of weak anchoring, microtubules grow in all directions, but occasionally, as they encounter the membrane, their direction may be transiently tangent to the membrane. The typical distance between microtubules and membrane ranges from 50 to 250 nm (Fig 2A) according to the shape. Even if weak anchoring allows microtubules to grow through all the cell volume, we find that such weak interaction with the membrane is enough to elicit the existence of cortical microtubules. Therefore, the three-dimensional nature of our model helps us demonstrate that strong anchoring is not required for the presence of large populations of cortical microtubules in plant cells: the directional persistence, together microtubule growth mode, can cause such sub-cellular localization.

As the microtubules tend to stay at close to the membrane, they also bundle, with the proportion of tubulin in bundles varying from 30% to 75% (Fig 2B). The ability for the microtubule network to generate a spontaneous bundled structure is consistent with previous models constraining microtubules to the surface. Strikingly, this effect is also present in the case of weak anchoring.

### Strong anchoring decreases microtubule number and length, and increases microtubule bundling

Independently of the encounter rule, weak anchoring strength increases the total number and the size of microtubules (by about 20%, in length and in number) when compared to strong anchoring. Weak anchoring also yields less bundling (reduction of about 20%). A likely explanation is that a weak anchoring to the membrane allows microtubules to “escape” inside the cell, thus diminishing the encounter probability. Consequently, microtubules weakly bound to the membrane have more space to grow and are less subject to shrinkage-induced collisions or bundling with other microtubules (Fig 2B).

### The number and length of microtubules, as well as the proportion of microtubules in bundles, are relatively independent of cell shape

**Fig 3:**
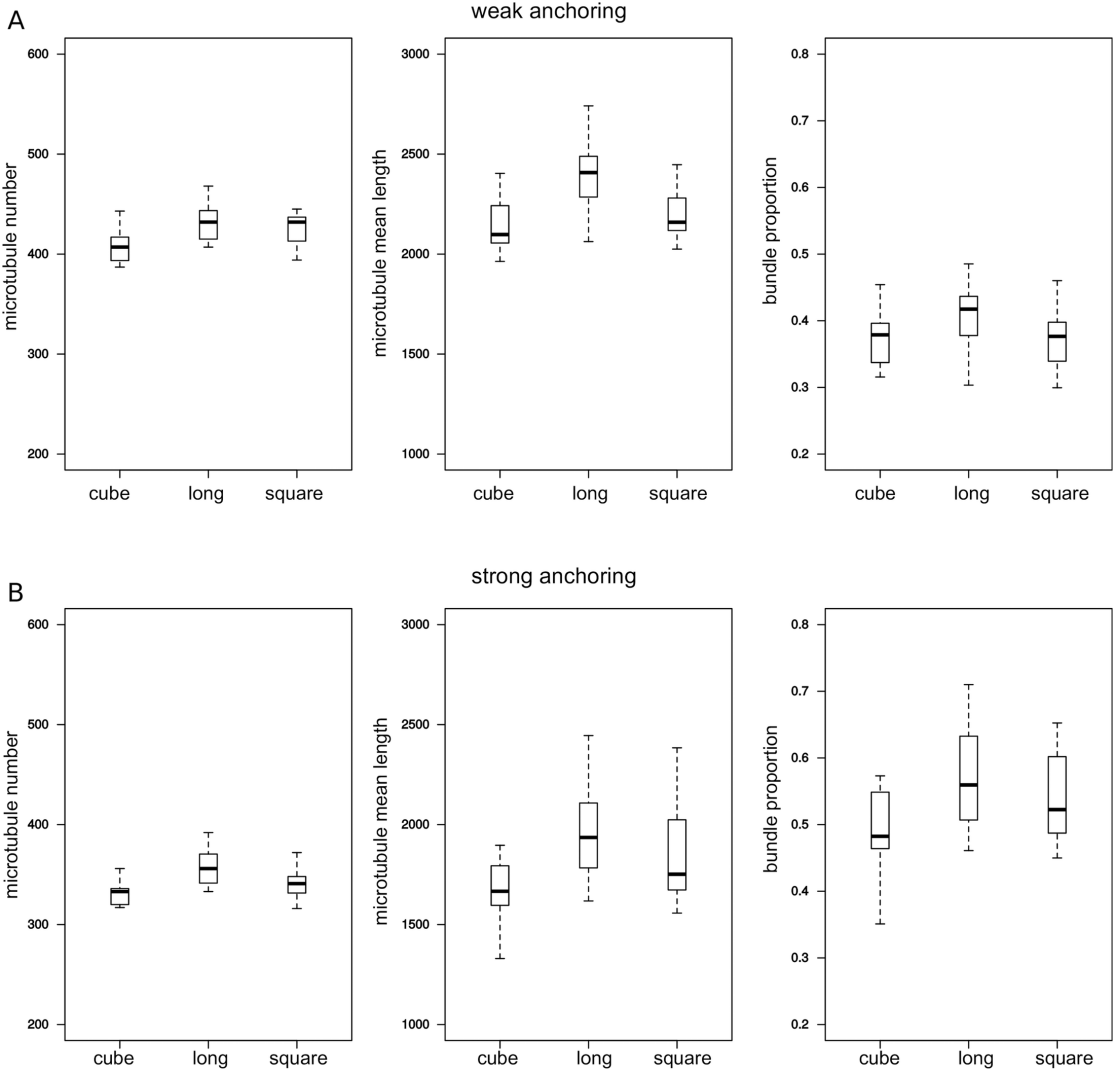
Influence of global shape on three properties of the network. Data for sharp shapes: cube, square, and long (Fig 1). From left to right: number of microtubules, mean microtubule length, and proportion of tubulin in bundles. Top row: weak anchoring; bottom row: strong anchoring. Model parameters have the default values (see Methods).

Next, we used our model to determine the consequences of changing cell shape on global properties of the network. We simulated the network in three main sharp shapes represented on Fig 1A. The results from the simulations indicate that these shapes only have a small effect on the number of microtubules (less than 10% difference), on the length of microtubules (5 to 15% difference) and on the proportion of bundles (less than 15% difference). Overall, elongated cells have more microtubules, more bundles, and longer microtubules, while cubes show the lower values (Fig 3). A likely explanation is that microtubules are more likely to follow the long axis in the long shape (see below) so that they are less affected by cell boundaries.

### Microtubule array anisotropy is influenced by cell curvature but not by global shape

**Fig 4:**
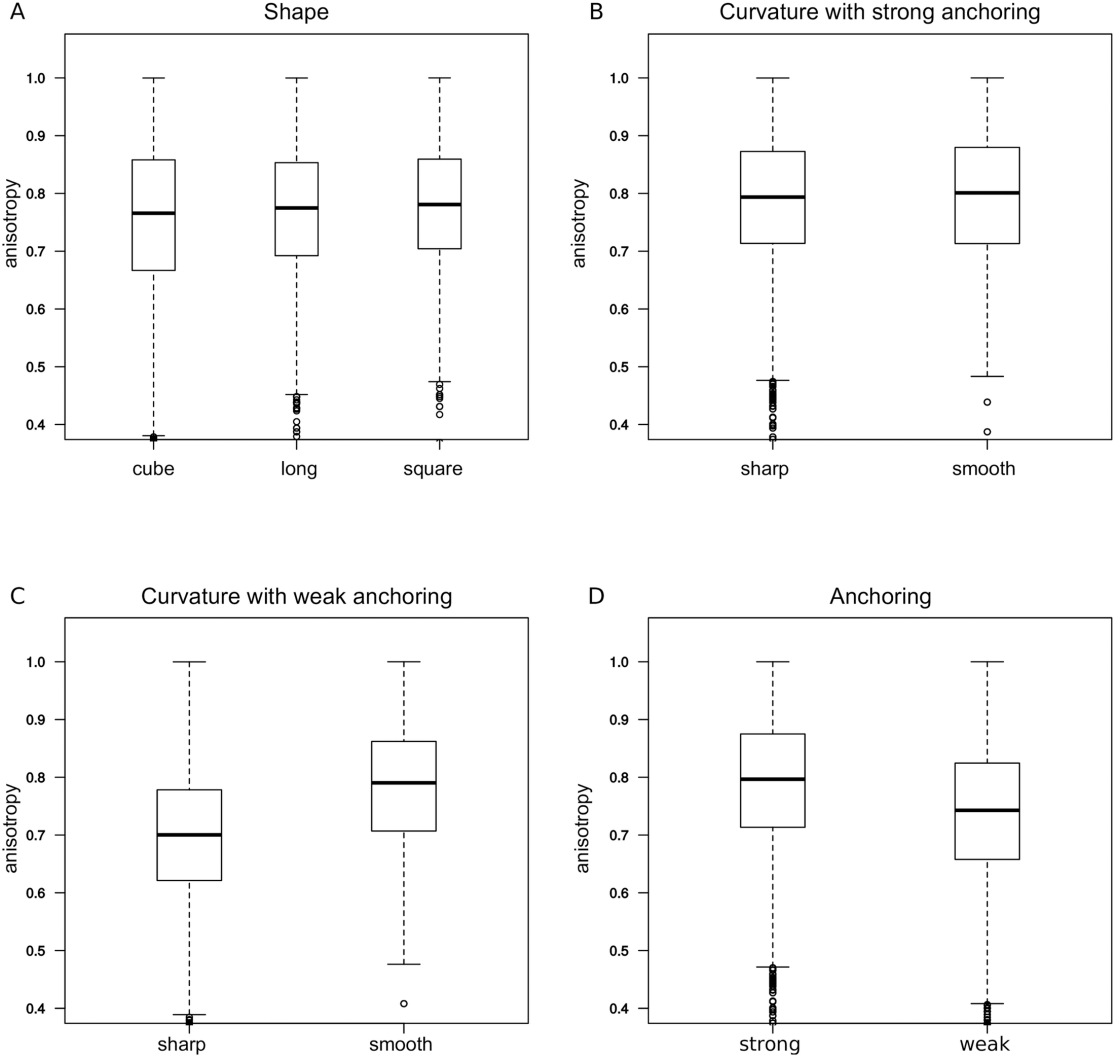
Anisotropy of the network. (A) Effect of global shape: Anisotropy for the three shapes (sharp/smooth and weak/strong anchoring were pooled together). (B) Effect of curvature: Anisotropy for sharp shapes vs. smooth shapes with strong anchoring. (C) Effect of curvature: Anisotropy for sharp shapes vs. smooth shapes with strong anchoring. (D) Effect of anchoring strength: Anisotropy for all shapes (pooled). In all panels, the data are pooled except for the parameter that is varied in the subfigure; model parameters have the default values (see Methods).

We also analyzed the effect of cell shape on the microtubule array anisotropy, averaged over the cell (see Methods); this is quantified with an order parameter with values between 0 and 1. As microtubule array anisotropy is skewed towards low values (found inside the cell) we used a non-parametric test based on ranks for statistical comparisons. Consistently with its effect on bundling (see above), we found that anchoring strength slightly affects the anisotropy of the microtubule arrays. Weak anchoring decreases the anisotropy of the network by about 10% (Fig 4C).

The type of cell shape did not appear to influence the global anisotropy of the microtubule network (Fig 4A). This is interesting as it suggests that different cell types with various shapes do not require differential regulation of the network in order to maintain the anisotropic properties of their CMT arrays.

However, smooth and sharp shapes differ significantly in anisotropy of the microtubule network (by 10%, *p* < 0.001); more curved cell edges in sharp shapes correspond to a lower anisotropy (Fig 4B). Larger surface curvature induce more “heads-on” collisions when microtubules grow nearby, leading to a smaller microtubule density, as they become more distant from the surface (Fig 2A); this would lead to more spatial variations in the orientation of microtubules and hence lower anisotropy.

### The average microtubule orientation is strongly influenced by cell shape

In order to determine how the shape of the cell influences the global orientation of the network, we measured the distribution of orientations along two opposite faces of the cells. We chose two arbitrary faces for cube shape, the largest faces for square shape, and two of the largest faces for the long shape; accordingly, these two opposite faces are among the two largest faces in area for the shape of interest. An angle of 0° corresponds to the long axis in the case of the elongated cell. Angles of 90° or -90° correspond to directions perpendicular to that axis.

**Fig 5:**
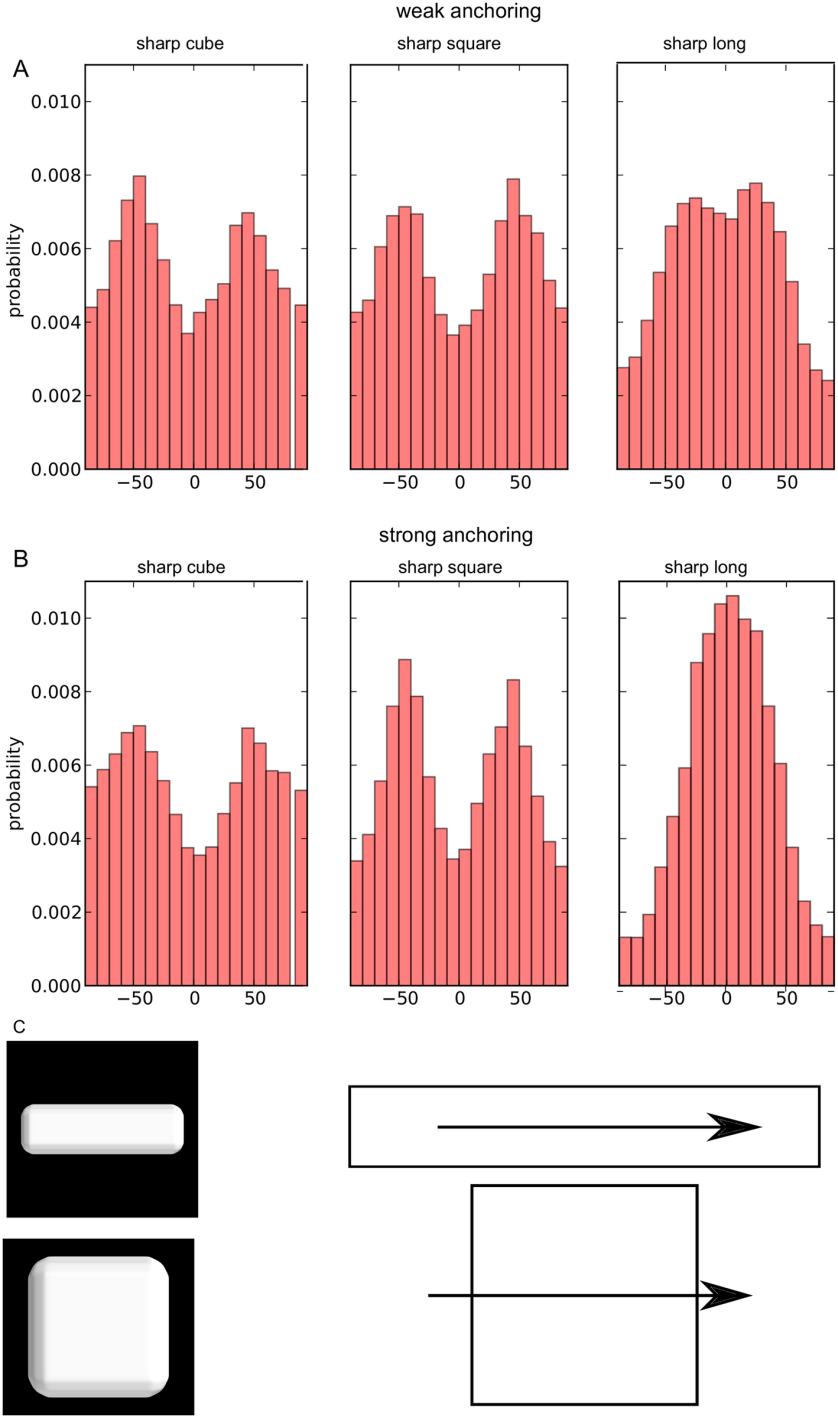
Orientation of the network. Distribution of the orientation of unit vectors (local orientation of microtubules) located close to the top and bottom faces. Top and bottom faces refer to the largest faces of the square shape represented in C, and to arbitrary opposite large faces of the cube or the long shapes. The reference angle 0 corresponds to the longest axis of the long cell and is parallel to one of the edges of the top face in the two other cases. (A,B) Distribution of orientations for sharp cube, sharp square, and sharp long shapes, as labeled in Fig 1. (A) Weak anchoring and (B) strong anchoring. (C) Arrow showing the reference for angle measurement for the long shape (top) and for the square and cube shapes (bottom). The shapes are not at the same scale. Model parameters have the default values (see Methods).

First, we observed that in the case of square shaped faces, most of the microtubules align along the cell face diagonal, i.e. the longest path (Fig 5). This occurred whatever the anchoring strength and the shape of faces at the side. Second, we observed a strong correlation between the axis of the cell and microtubule orientation, and this correlation is the highest for the elongated cell: The microtubule distribution is always either maximal or minimal at an angle of 0. This effect is higher in the case of strong anchoring, whereas in the case of weak anchoring, secondary peaks show that the diagonals are also overrepresented. These results indicate that the microtubule network is able to read the longest axis of the cell and orient toward that axis, by default. Interestingly, cortical microtubules become longitudinal in hypocotyl cells when growth stops [5, 55, 56], suggesting that they may adopt their configuration by default in that situation.

### Small external directional bias is sufficient to affect the orientation of the whole network

**Fig 6:**
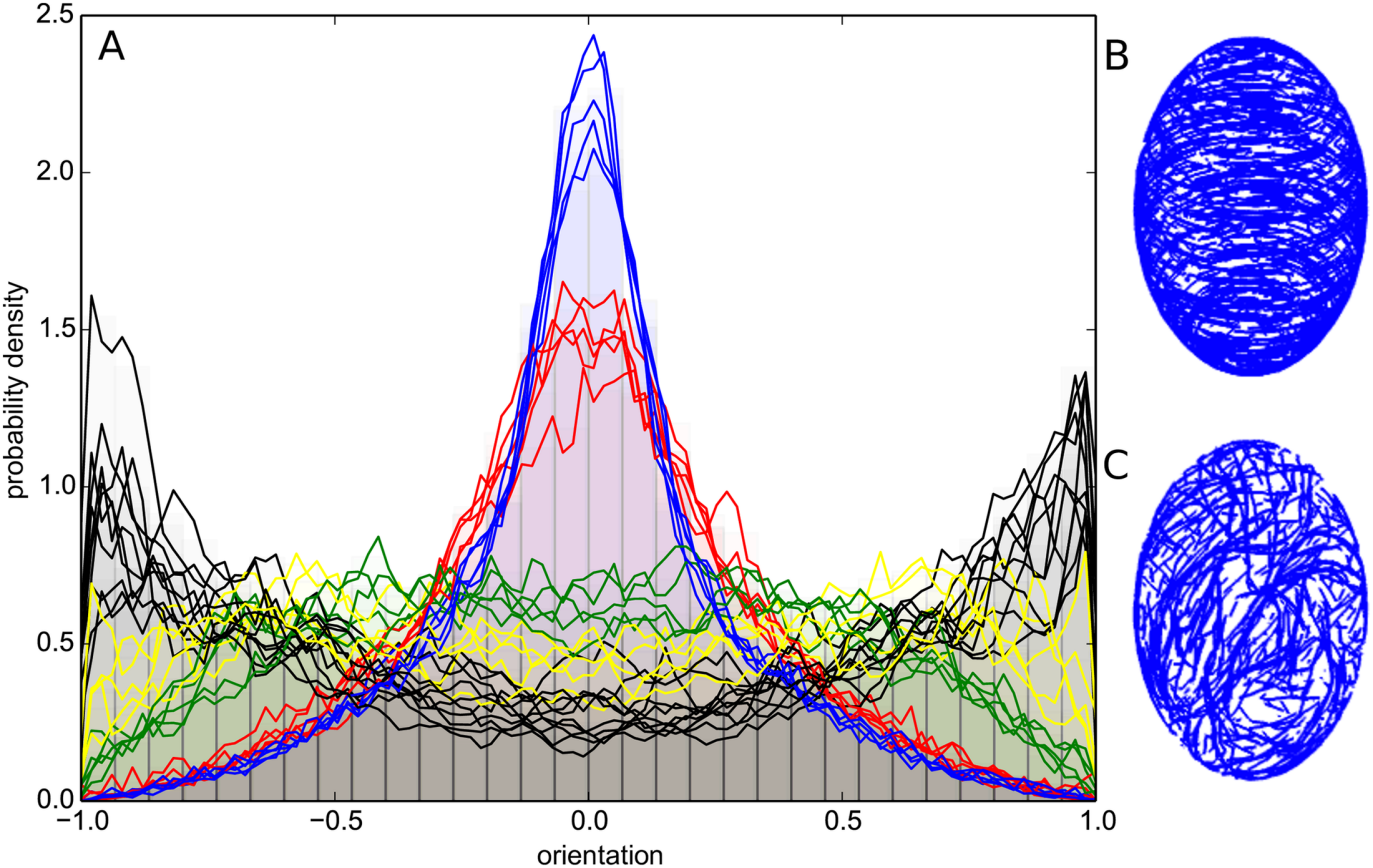
Influence of directional cues on the global orientation of the network. Simulations in an ellipsoidal cell with a circumferential cue. (A) Histogram of the scalar product between the unit vectors (defining microtubules locally) and the long axis of the ellipsoid. Black: no directional cue. Colors: directional cue with influence on the direction. The strength, *b*_*d*_, of the signal is: 0.1% (yellow), 0.2% (green), 1% (red), and 2% (blue). (B) Simulation result for a weight of 2%, also see S2 Video. (C) Simulation result with no directional cue. Model parameters: *n*_*p*_ = 2.4 ⋅ 10^−7^, *n*_*s*_ = 10^−3^, *α* = 40°, weak anchoring.

Next, we investigated the robustness of microtubule network. Microtubule arrays entirely reorganize during cell division [57]. Light and hormones can also completely reorient the microtubule network within minutes [33, 34, 58], suggesting that the constraints on microtubules array must not be too strong to allow such rapid reorganization in vivo. We tested whether our model provides such adaptability, using the case of external, directional cues (Fig 6 and S2 Video).

We investigated the effect of an external cue, assuming that, as microtubules grow in the cell, their growth direction is biased toward the direction of cue, with a specific weight. We used an ellipsoidal cell with a circumferential cue, which is orthoradial to the long axis of the cell. Such a cue could, for instance, be related to cell polarity (vector along the long axis) or could be a proxy of mechanical tension [19]. In the absence of the cue, microtubules are on average parallel to the main axis of the cell. Strikingly, our simulations indicate that even a very low bias (0.1%) could disrupt the main orientation of the network. When the weight reaches 1% or more, microtubules massively reorient toward the direction of the cue. Interestingly, the transition between the longitudinal and circumferential orientation occurs when the bias is comparable to the directional noise that is used to account for the persistence length of microtubules. This reinforces the idea that fluctuations of microtubules and the interactions between them lead to alignment with the long axis of the cell and that the small cue is sufficient to counteract these fluctuations and influence the orientation of microtubules. Accordingly, we found that anisotropy increases from no cue to a weight of about 1% and then seems to saturate (S1 Fig).

These results indicate that, despite an apparent robust organization, the microtubule network remains extremely sensitive to directional cues. As such, it is capable of reading slight directional cues and generating an ordered array in that direction. This ability to read directional cues is probably linked to the self-enhancement of microtubule orientation through their interactions: As more microtubules orient toward a direction, they prevent growth of microtubules in the perpendicular direction.

Our simulations indicate that the network should exhibit two behaviors concerning its orientation. When no external directional cue is present, the network orients toward the main axis of the cell, and generates a polarity that is a direct reading of the global shape. When a directional cue is present, the network reorients so as to emphasize the direction of the cue.

## Discussion

### A model for microtubules growing in the cell volume

The impact of external cues on the microtubule network has been extensively characterized in experiments. However, because real cells and tissues are never really devoid of external cues, the behavior of the microtubule network by default in a plant cell remains an open question. Our work provides some clues to address this question, by taking into account the 3D shape of the cell and by using a minimal set of parameters for microtubule behavior. For instance, the model with weak anchoring does not require a specific rule for the crossing of microtubules (unlike when microtubules are confined to the cell surface), because microtubules naturally cross each other if they are farther than their diameter. We found that microtubule directional persistence largely determines the subcellular localization and orientation of the microtubule network in various cell shapes. We also identified parameters that seemed relatively insensitive to cell shape, such as microtubule length or number. Last, we found that microtubule dynamics yields at the same time stable orientation and high sensitivity to directional cues, even when such cues go against the default orientation. Altogether, this provides a conceptual framework to dissect the exact contribution of microtubule regulators to the microtubule network organization, in relation to 3D cell shape.

### Cortical localization is an emerging property of microtubule directional persistence and dynamics in convex cells

Based on TEM images where microtubules are often seen very close to the plasma membrane, it is assumed that anchoring of microtubules to the plasma membrane is relatively strong. However it remains unknown how such anchoring would be mediated [52]. Many biochemical studies have been performed to extract proteins that would link the plasma membrane to cortical microtubules, and so far the only published candidate is a phospholipase [59], for which no follow-up results have been obtained to the best of our knowledge. Other links have been put forward, such as CLASP [10] or CELLULOSE SYNTHASE INTERACTIVE PROTEIN 1 (CSI1) [53, 60], but they might represent rather indirect regulators of the link between microtubules and plasma membrane. Does this mean that microtubule could be cortical without any anchoring module? Our results suggest that in most of the measured cell shapes, microtubules do not need to be strongly connected to the membrane to remain cortical. This prediction implies that modulating anchoring would affect self-organizing properties tangentially to the cell surface, rather than modulating the density of microtubules inside the cell. This would allow molecular regulators to modify the microtubule organization without directly affecting the rate of cellulose deposition.

### A high curvature increases anisotropy

In our simulations, we observed that anisotropy varied according to curvature of face-face contacts of the cell, which is an emerging property of the weak anchoring mode. Cells that have sharper edges exhibit decreased array anisotropy compared to smoother cells. This result is interesting, as, for instance, epidermal cells possess edges with different curvatures [10]. Similarly, in the L1 layer of the shoot apical meristem, the outermost wall has a stronger curvature than all the walls separating the cell from its neighbors. Pavement cells also display a broad range of curvature values. Based on our results, higher anisotropy would be produced in L1 cells without the need for specific regulation.

Such results are difficult to account for in models with microtubules constrained to surfaces embedded in 3D, in which for instance additional hypotheses linking curvature and catastrophe rate are implemented [10]. Nevertheless, our results do not preclude microtubule associated proteins from additionally modulating microtubule dynamics according to curvature or to other membrane-localized cues [52]; we only account for the default behavior of microtubule networks according to surface curvature.

Similarly, changes in the curvature of the epidermal wall can occur through changes in internal pressure, which in return influence the anisotropy of the network [61]. An increase in the microtubule organization could be a result of an increased pressure, that would increase the curvature of the epidermal cell. Further experimental work is required to investigate the role of curvature on microtubule behavior and its relation to mechanical stress in the epidermis.

### Influence of shape on the global directionality of the network

Our simulations show that the cell aspect ratio has an important impact on the global orientation of the network. The predicted default behavior of microtubules is their alignment parallel to the long axis of a cell, due to the directional persistence of microtubules. This is in agreement with previous models with microtubules confined to surfaces embedded in 3D [11, 51], where the default orientation is longitudinal for long cylinders.

This default state was observed with microtubules polymerizing in vitro inside elongated 3D chambers [11]. In slowly growing cells of the hypocotyl, microtubules are oriented along the long axis of the cell, whereas microtubules are circumferential in rapidly elongating cells [55, 56]. Our model suggests that directional cues are needed to avoid this default orientation is growing cells. At the boundary between the shoot apical meristem and the primordia, cell division leads to cell shapes that are elongated along the axis of the boundary; our model predicts that microtubules will be oriented along the same direction by default, amplifying their response to mechanical stress [19].

### A weak directional bias is sufficient to change the orientation of the microtubule network

In this study, we show that the microtubule network is oriented by default along the longest axis of the cell. However, microtubules in plants often show supracellular orientation, independently of cell shape, a behavior that has been ascribed to tissue-level signals, notably mechanical stress [18–20]. Moreover, It has been demonstrated that inside the cell, microtubules orientation is coupled to polarity markers such as proteins from the PIN FORMED and RHO OF PLANTS families [62, 63]. Simulations have assessed how localized membrane heterogeneity could result in a biased orientation of the microtubule network [10, 41]. In this study, we show that a weak directional cue influencing microtubule growth rapidly modifies the orientation of the network towards the direction of the cue. This cue could be due to mechanical stress, hormone gradients [22], or to cytoplasmic streaming [23, 24], for instance. As such, the network behaves as a sensor translating an external directional information into a structural polarity inside the cell. The coexistence of a default orientation and a strong ability to reorient could shed a new light on orientation changes in cells. Changes in microtubule orientation need not be always related to specific regulation but may also be related to the arrest of signals and the return to the default state. This could be occurring in the shift from transverse to longitudinal orientation in hypocotyls responding to light or hormones [33, 34, 58].

### Perspectives

The shape of the cell has little influence on mean length, number of microtubules or bundles proportions. In addition, anisotropy of the network is not highly correlated to changes in global cell shape. This prediction of a robust network suggests that plant cells do not need specific regulations to compensate for their great variations in shapes. Accordingly, the microtubule network appears as a good polarity system, with a default orientation and a high sensitivity to directional cues. It was recently shown that, for global polarity to emerge in a tissue, an important requirement is the existence of internal cellular polarity [64]. In this work we show that the microtubule network is suited for such a requirement.

Overall, microtubules and associated proteins form a complex self-organizing system that is difficult to comprehend without resorting to models. The results obtained here demonstrate that our three-dimensional model provides a framework to test hypotheses on the regulation of the microtubule cytoskeleton in plant cells.

The model given here is only the beginning to a more complete analysis. We have not yet incorporated microtubule severing [44, 58], microtubule branching [26, 27], nucleation at the nuclear envelope [28], or the possible effects of connections between cortical microtubules and cellulose fibrils outside of the cell as mediated by the cellulose synthase complex [65]. Severing in particular has been shown to be key to microtubule reorientation [44] following mechanical signals [66]. We also have not included limiting levels of tubulin [51], which could affect overall microtubule number. Altogether, we expect our model to help progress in understanding how microtubule self-organization integrates directional cues with three-dimensional cell shape and how microtubule-associated proteins modulate this integration.

## Methods

The dynamical microtubule model was implemented in C++. The code is available from https://gitlab.com/slcu/teamHJ/vincent/microtubule_simulations. Simulations were performed on Intel/AMD desktop computers running Debian and Ubuntu operating systems.

### The microtubule network

Microtubules were coded as 3D multi-segment vectors of constant length. A ring of tubulin of the length of a dimer is represented as a unit vector in the simulation. Microtubule growth in the model occurs by adding one vector element at the plus end of the microtubule, at the position of the end of the last vector. In plants, microtubules are considered to be mostly static, their growth and shrinkage are the result of treadmilling processes. To code for microtubule directional persistence (which relates physically to bending stiffness), the direction at which a new vector is added to an existing microtubule changes by a small random amount. Microtubule shrinkage occurs at the minus end by removing the first vector from the list. The main model parameters are shown in Table 1.

**Table 1:** Main parameters for microtubule dynamics.

Parameter	Nucleation frequency	Shrinkage probability	Random direction	Encounter angle	Anchoring strength	Bias strength
Label	*n*_*p*_	*n*_*s*_	*r*_*d*_	*α*		*b*_*d*_
Default value	2.4 ⋅ 10^−7^, 4.7 ⋅ 10^−7^, and 7.1 ⋅ 10^−7^ per unit surface per time step	0.001 per nucleation site per time step	0.025	40°	strong or weak	0

### Time and space units

Typically, a cell has a width of several micrometers, and we take the unit of length as 8nm, the height of a ring of tubulin. Considering a measured speed of growth at plus ends of 3-5 *μ*m/min [67], a simulation time step is approximately 0.1 to 0.2 s. A typical simulation of 10000 time steps thus represents 15 to 30 min of real time.

### Nucleation and minus end behavior

The microtubules are nucleated on the cell surface [25–27] at a constant rate. The default value is *n*_*p*_ = 4.7 ⋅ 10^−7^ per time step per unit surface, corresponding to 1 to 2 nucleation events per time step, or 5 to 20 per second. Once nucleated, the microtubules do not immediately shrink. At each of the time steps that follow nucleation, a microtubule has a probability *n*_*s*_ to begin shrinkage. Once a microtubule has started to shrink, one vector is removed from the minus end at each time step.

### Plus-end growth

#### Default growth

At each time step, a new vector is added to the plus end. In the absence of interactions with the membrane or with neighbouring microtubules, the new direction is that of the previous vector (tubulin), modified by a random direction. The new unit vector is computed as

$$\begin{array}{c} r_{n+1}=\frac{\left(1-r_{d}\right)r_{n}+r_{d}u}{\|\left(1-r_{d}\right)r_{n}+r_{d}u\|}, \end{array}$$ (1)

where **r**_*n*_ is the previous unit vector, **u** is a random unit vector, and ‖ ‖ is the standard Euclidian norm.

At lower order in *r*_*d*_ (which is a small parameter), the correlation between two consecutive vectors take the form $<r_{n+1}\cdot r_{n}>=1-r_{d}^{2}/4$ and should be equal to 1 − 1/2*ℓ*/*p* where *ℓ* is the length of one vector and *p* is the persistence length [68]. Therefore, the default value of the random direction parameter *r*_*d*_ = 0.025 corresponds to a persistence length $p=2\ell /r_{d}^{2}$ of 26 *μ*m. This is in the range of values for persistence length measured in vivo, 20 *μ*m [69] to 30 *μ*m [70], which is much smaller than those found in vitro [71, 72], likely due to the action of proteins interacting with microtubules.

#### Directional cue

In the presence of directional cue, the new vector unit vector is computed as the weighted average of its default direction and of the direction of the cue (unit vector **b**) weighted by *b*_*d*_:

$$\begin{array}{c} r_{n+1}=\frac{\left(1-r_{d}-b_{d}\right)r_{n}+r_{d}u+b_{d}b}{\|\left(1-r_{d}-b_{d}\right)r_{n}+r_{d}u+b_{d}b\|}. \end{array}$$ (2)

The directional cue was only implemented for the ellipsoidal shape, where it was assumed to be circumferential (orthoradial) to the axis of revolution (long axis).

#### Interaction with a pre-existing microtubule

When a plus end is closer than 25 nm to another (pre-existing) microtubule, it follows one of two behaviors according to the angle between the two microtubules:

- if the angle is smaller than a threshold *α*, the microtubule begins zippering, and its direction becomes permanently that of the pre-existing microtubule,
- if the angle is larger than *α*, the microtubule begins to shrink at the plus end (catastrophe event). It will continue shrinking by one vector at each of the following timesteps until it disappears.

*α* was set to 40° following experimental observations [9].

#### Growth near the surface

When a microtubule reaches the plasma membrane, it follows one of two behaviors depending on the interaction strength with the membrane.

- Strong anchoring—The microtubule growth is limited to the local plane tangential to the surface, which is calculated at each time step. The new direction will only have two degrees of freedom, corresponding to the projection of the unit vector (Eqs 1 and 2) on the tangential plane. For this reason, microtubules remain at the surface of the cell. This is equivalent to previous models constraining microtubules on the cell surface [10, 11, 41, 51].
- Weak anchoring—Microtubules may grow inside the the cell. If a microtubule reaches a distance of 10 nm from the membrane, the angle between the microtubule and the local tangential plane is calculated. We use the same rule as for the interaction with another microtubule. If the angle is greater than *α*, then the microtubule starts shrinking at the plus end; it will continue shrinking by one vector at each of the following timesteps until it disappears. If the angle is smaller than *α*, then the new vector is computed according to Eqs (1) and (2). If the vector points outside the cell then it is re-computed (with new random choices of **u**) until it points inside the cell or tangentially. Thus, the microtubules remain encased in the cell at all times.

### Cell shape and interaction with the membrane

The cell contour is described with a triangular mesh. Each vertex is endowed with the information of the vector normal to the surface, which is used during the simulation to calculate a local approximation of the tangential plane. It is possible to add other informations at the vertex level that can be read during the simulation and serve as extrinsic input. Inputs can be scalars or tensors. The distance from any point in space to the membrane is calculated using the nearest point on the surface. At this point, the membrane is approximated by the plane perpendicular to the normal of the mesh. The distance between a tubulin ring (unit vector) and the membrane is calculated as the shortest distance between the endpoint of the vector and this plane. A collection of standard cell shapes was generated for our simulations (Figs 1 and 6). The main shapes were constructed starting from square parallelepipeds of dimensions 8.8*μ*m×8.8*μ*m×8.8*μ*m (cube), 9*μ*m×9*μ*m×4.7*μ*m (“square”), and 4.8*μ*m×4.8*μ*m×15.6*μ*m (“long”), which are comparable to typical plant cell dimensions. Then the meshes were smoothed so that the minimal radius of curvature was 1.3*μ*m and 4.7*μ*m for sharp and smooth shapes, respectively, which roughly spans the range 0.5-5*μ*m measured in plant roots [10]. The ellipsoid shape is an ellipsoid of revolution around the long axis; the short and long axis have dimensions of 10.3 *μ*m and 16.8 *μ*m, respectively.

### The simulation process

The simulation progress is made through discrete timesteps. At each timestep new vectors are added and vectors are removed from the simulation space according to the rules specified in the previous subsections (Nucleation and minus end behavior; Plus end growth). Collision tests are performed so as to implement the different growth or shrinkage rules. In order to increase the simulation speed, space is divided into subelements and the vectors are identified according to the subelement to which they belong, which diminishes the number of particles involved in the collision test (locality-sensitive hashing [73]).

### Visualization

To visualize microtubule density and orientations an image is created using a matrix of resolution (*r*_*x*_, *r*_*y*_, *r*_*z*_). *r*_*z*_ is larger than *r*_*x*_ = *r*_*y*_ by typically a ratio of 10 to 1, which mimics the anisotropic resolution of a confocal microscope. The simulation space is then screened. When a vector is located inside a cube of the matrix, the value of this cube is incremented by one, and the immediate neighbouring cubes are incremented by a lower number (typically 0.3). At the end of the process, a stack is formed where microtubules appear as blurred intensity signals. One can either visualize each sub-image from the stack by moving along the *z* axis, or create a projection that sums the matrix along the *z* axis.

### Quantifying anisotropy of microtubule arrays

We used the standard nematic order parameter to quantify anisotropy. The space is subdivided into cubes of arbitrary size, typically segmenting the structure into circa 27 cubes (S2 Fig). Segmenting the structure into 216 smaller pieces gives similar trends, with globally higher anisotropy values. All tubulin ring directions are extracted as a 3 × *N* matrix, *D*. We then compute the square symmetric (3 × 3) matrix *M* = *D*^*T*^ ⋅ *D*/*N*, where ^*T*^ stands for the transpose. *M* is diagonalised, yielding three eigenvalues λ_*i*_, *i* ∈ 1, 2, 3. Local anisotropy, A, of the microtubule array in each cube of space is defined as

$$\begin{array}{c} A=\sqrt{\frac{3}{2}}\frac{\sqrt{{\left(\lambda_{1}-\lambda_{m}\right)}^{2}+{\left(\lambda_{2}-\lambda_{m}\right)}^{2}+{\left(\lambda_{3}-\lambda_{m}\right)}^{2}}}{\sqrt{\lambda_{1}^{2}+\lambda_{2}^{2}+\lambda_{3}^{2}}};\;\lambda_{m}=\frac{\lambda_{1}+\lambda_{2}+\lambda_{3}}{3}. \end{array}$$ (3)

The value of *A* is such that 0 ≤ *A* ≤ 1. Anisotropy value is then computed as the average of the local value *A* over the whole cell.

### Statistics

The simulations were run 5 times for each parameter value or shape considered; to avoid artificial correlations between data, only the last snapshot was considered for further statistical analysis. As data for the three default values of *n*_*p*_ were statistically identical, they were pooled together for Figs 1–5. The plots were produced with the boxplot function of R: the boxes extend between the first and third quartiles, the segments in the box indicate the medians, and the whiskers are representative of extreme values.

## Supporting information

S1 Fig Influence of directional cues on the average anisotropy of the network. Simulations in an ellipsoidal cell with a circumferential cue. (A) Anisotropy as a function of the strength of the signal, *b*_*d*_. (B-E) Snapshots showing the microtubules in half the cell (the half to the back was removed for clarity). The strength, *b*_*d*_, of the signal is: 0.1% (B), 0.2% (C), 1% (D), and 2% (E), respectively. Model parameters have the default values (see Methods) except for the variable *b*_*d*_ and for weak anchoring.
(PDF)

S2 Fig Calculation of anisotropy. (A) Smooth “square” cell shape. (B) Microtubules. (C) Unit vectors are shown as small white arrows plotted at the center of each cube. (D) The matrix *M* corresponding to each cube (see Methods) is diagonalised; the red vectors point in the direction of the eigenvectors and the length of the red vectors is proportional to the corresponding eigenvalues.
(PDF)

S1 Video Typical simulation. Smooth “square” cell shape with the default parameter for microtubule dynamics and weak anchoring. The duration of the movie is 5000 timesteps.
(AVI)

S2 Video Typical simulation with external cue. Ellipsoid cell shape with the following parameters for microtubule dynamics: *n*_*p*_ = 2.4 ⋅ 10^−7^, *n*_*s*_ = 10^−3^, *α* = 40.1°, weak anchoring, *b*_*d*_ = 0.02; the duration of the movie is 5000 time steps. The microtubules are shown in half the cell (the half to the back was removed for clarity).
(AVI)
