# Supplementary figures and images for: The self-organization of plant microtubules inside the cell volume yields their cortical localization, stable alignment, and sensitivity to external cues
Source: PLoS Comput Biol. 2018 Feb 20;14(2):e1006011. doi: 10.1371/journal.pcbi.1006011 (PMC5834207; doi:10.1371/journal.pcbi.1006011)

**A**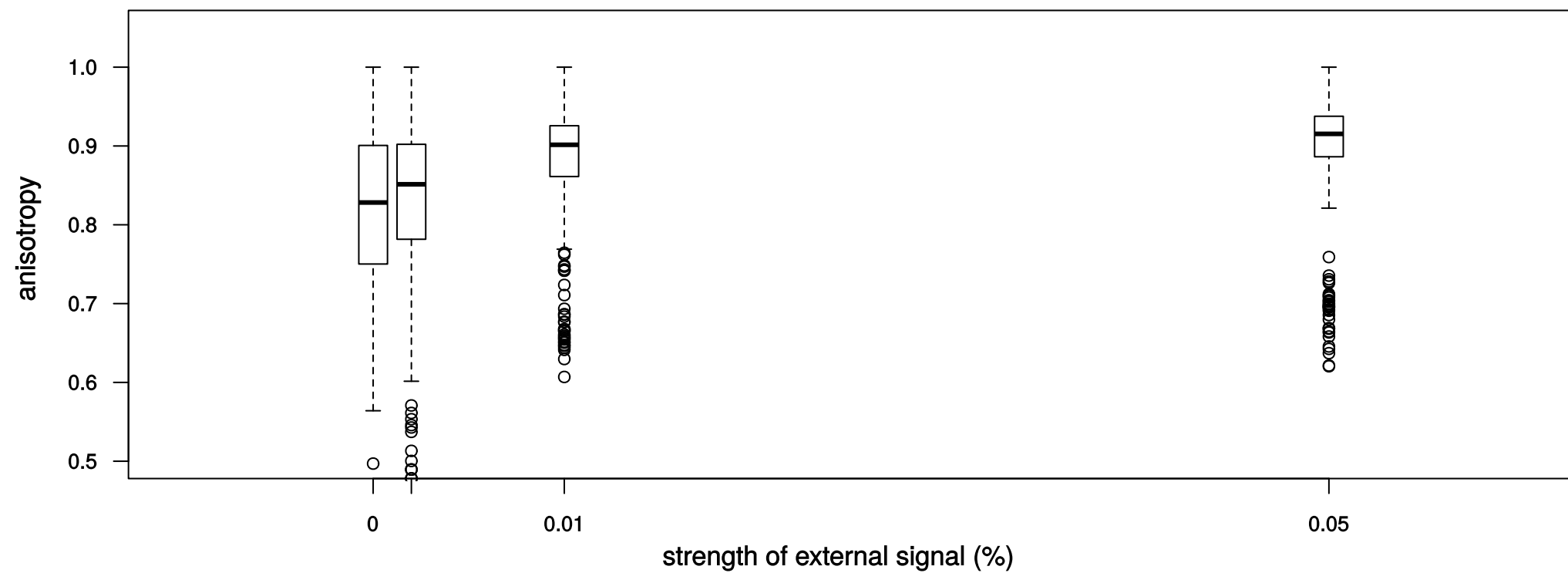**B**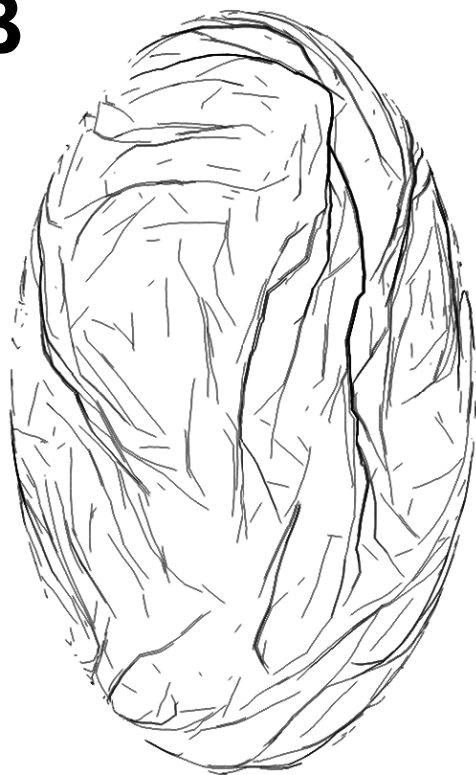**C**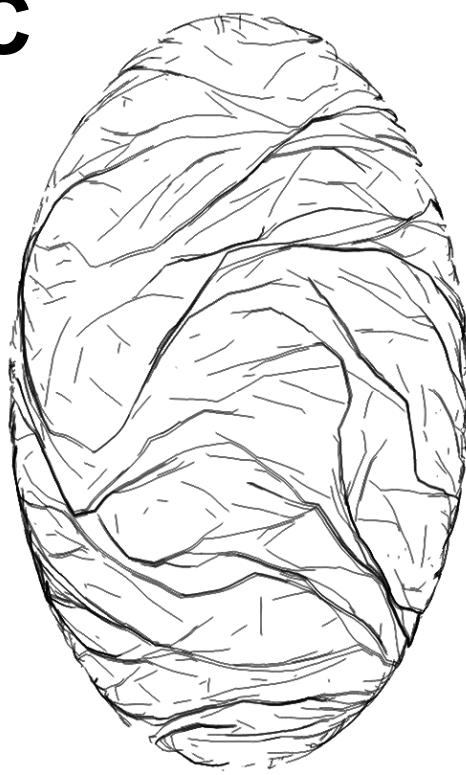**D**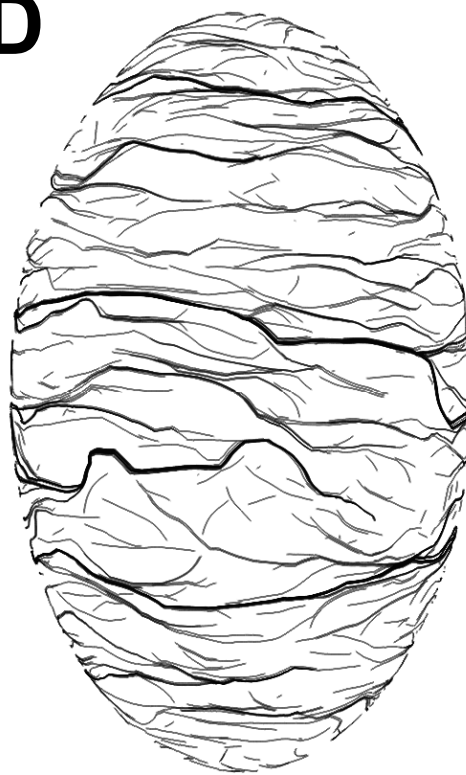**E**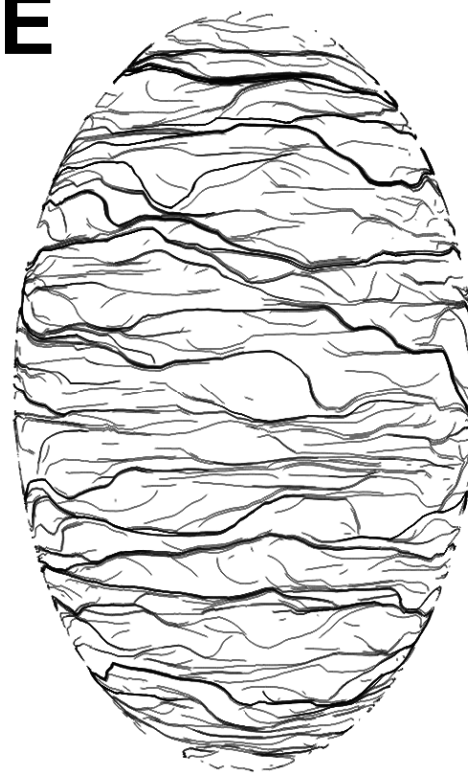

Supplement: S1 Fig — Simulations in an ellipsoidal cell with a circumferential cue. (A) Anisotropy as a function of the strength of the signal, bd. (B-E) Snapshots showing the microtubules in half the cell (the half to the back was removed for clarity). The strength, bd, of the signal is: 0.1% (B), 0.2% (C), 1% (D), and 2% (E), respectively. Model parameters have the default values (see Methods) except for the variable bd and for weak anchoring. (PDF) [file pcbi.1006011.s001.pdf]

A

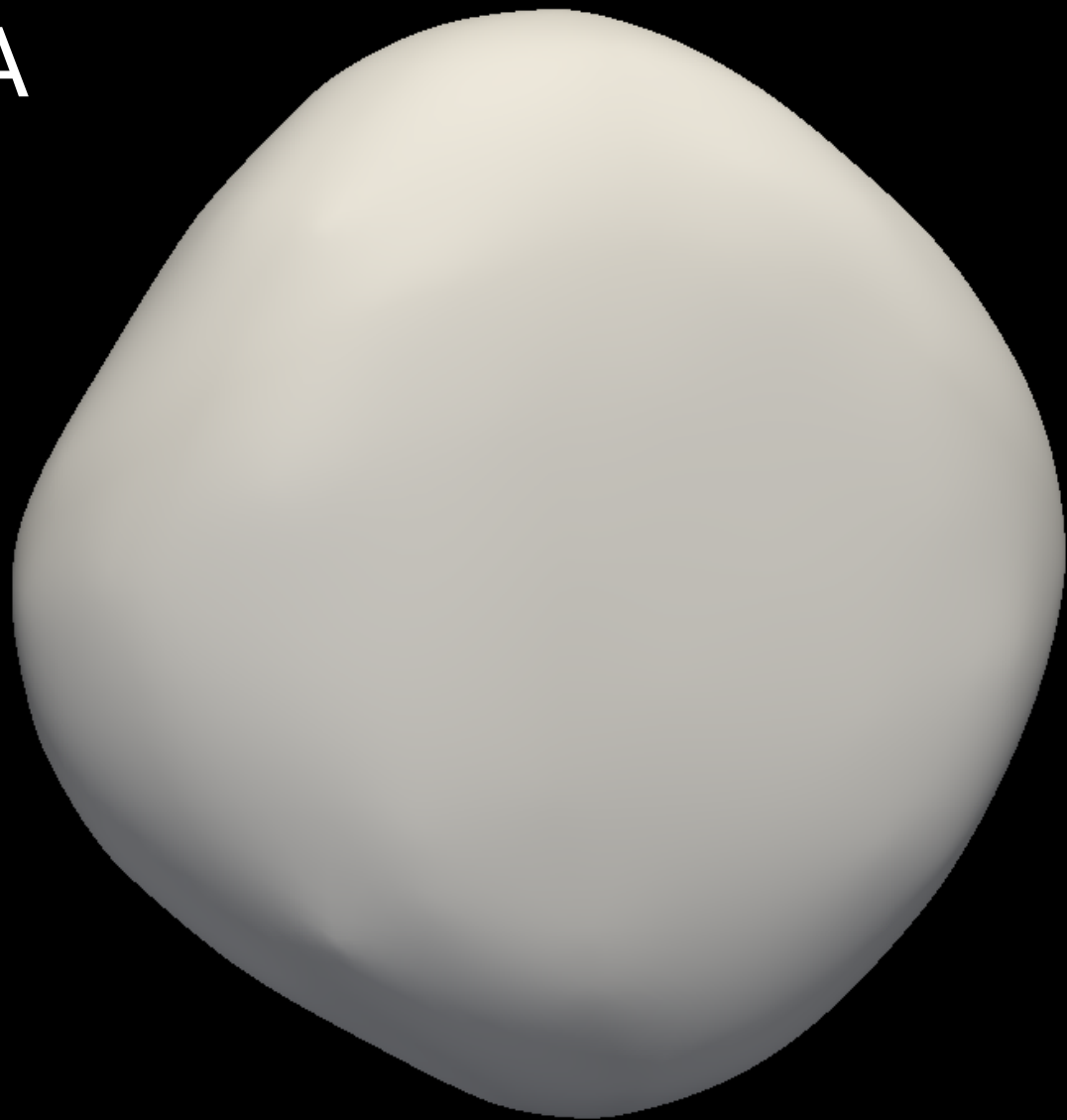

B

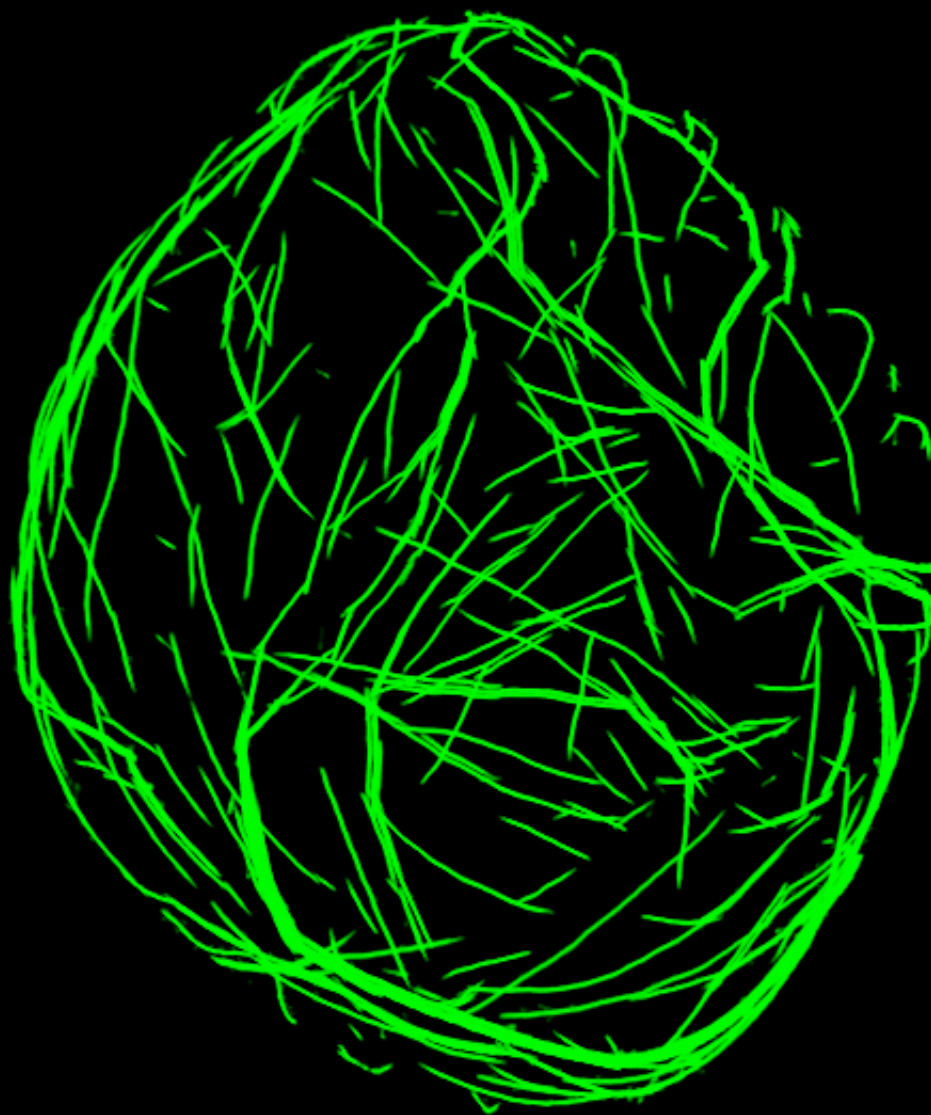

C

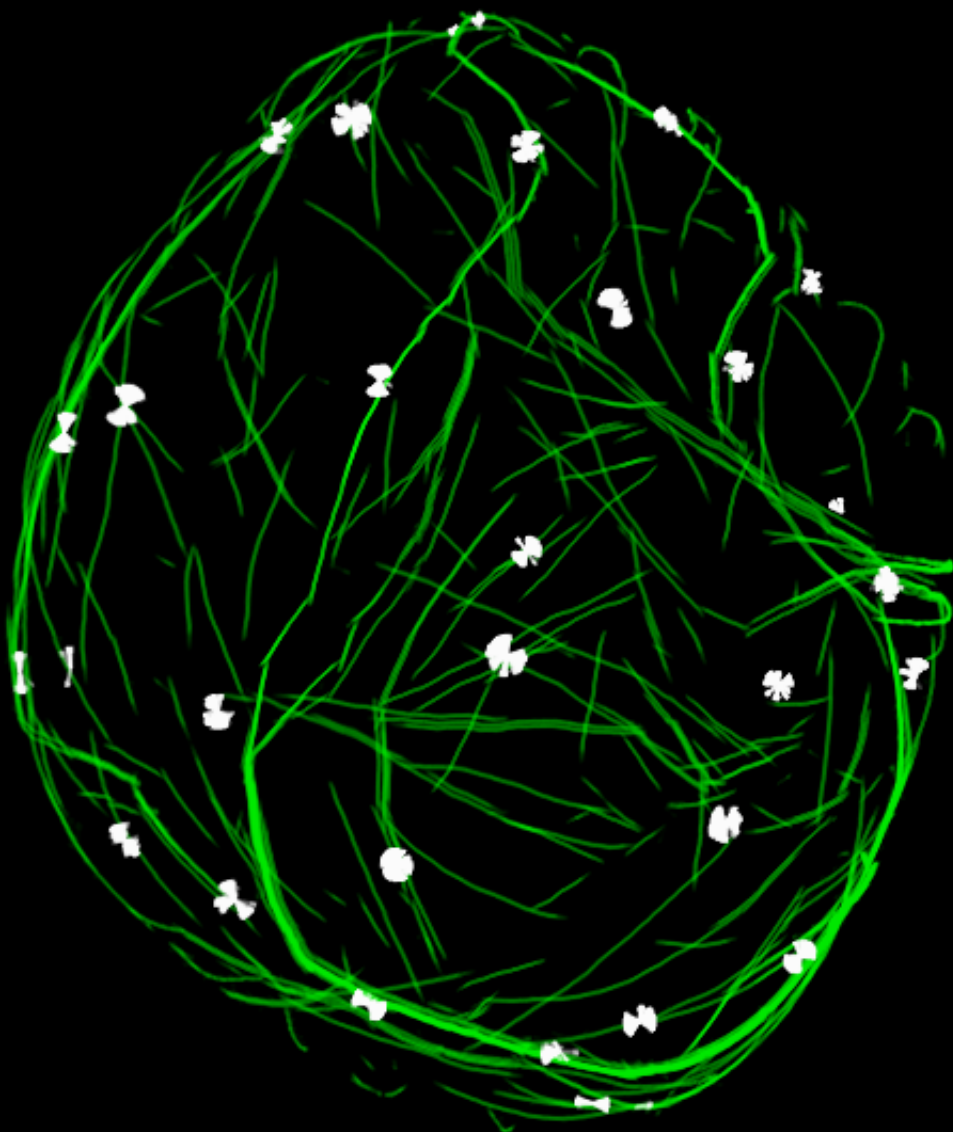

D

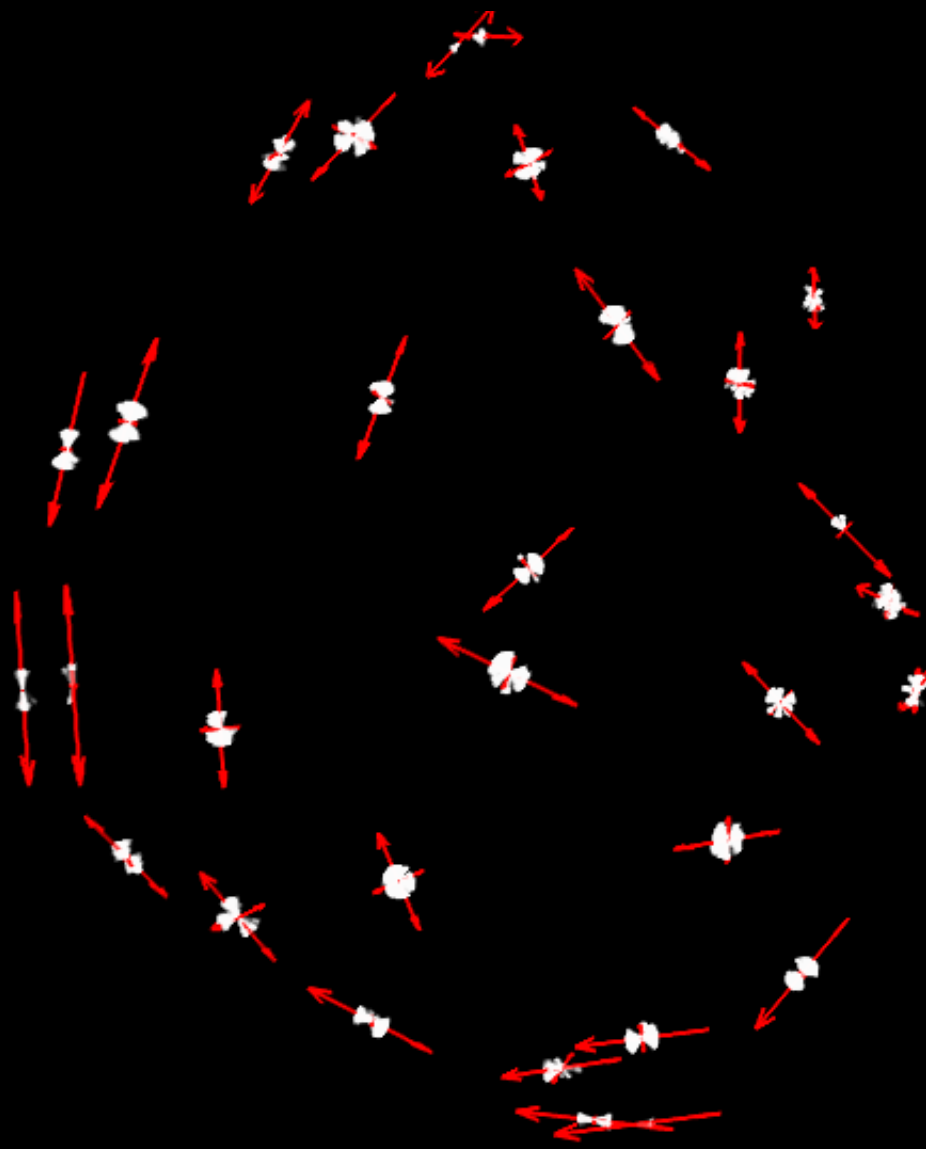

Supplement: S2 Fig — (A) Smooth “square” cell shape. (B) Microtubules. (C) Unit vectors are shown as small white arrows plotted at the center of each cube. (D) The matrix M corresponding to each cube (see Methods) is diagonalised; the red vectors point in the direction of the eigenvectors and the length of the red vectors is proportional to the corresponding eigenvalues. (PDF) [file pcbi.1006011.s002.pdf]
